# *miR-29a/b_1_* Regulates the Luteinizing Hormone Secretion and Affects Mouse Ovulation

**DOI:** 10.3389/fendo.2021.636220

**Published:** 2021-05-31

**Authors:** Yang Guo, Youbing Wu, Jiahao Shi, Hua Zhuang, Lei Ci, Qin Huang, Zhipeng Wan, Hua Yang, Mengjie Zhang, Yutong Tan, Ruilin Sun, Leon Xu, Zhugang Wang, Ruling Shen, Jian Fei

**Affiliations:** ^1^ School of Life Science and Technology, Tongji University, Shanghai, China; ^2^ Shanghai Lab, Animal Research Center, Shanghai, China; ^3^ Shanghai Model Organisms, Shanghai, China; ^4^ Department of Medicine, Ruijin Hospital, Shanghai Jiao Tong University, Shanghai, China

**Keywords:** *miR-29a/b_1_*, knockout, LH, ovulation, reproduction

## Abstract

*miR-29a/b_1_* was reportedly involved in the regulation of the reproductive function in female mice, but the underlying molecular mechanisms are not clear. In this study, female mice lacking *miR-29a/b_1_* showed a delay in vaginal opening, irregular estrous cycles, ovulation disorder and subfertility. The level of luteinizing hormone (LH) was significantly lower in plasma but higher in pituitary of mutant mice. However, egg development was normal in mutant mice and the ovulation disorder could be rescued by the superovulation treatment. These results suggested that the LH secretion was impaired in mutant mice. Further studies showed that deficiency of *miR-29a/b_1_* in mice resulted in an abnormal expression of a number of proteins involved in vesicular transport and exocytosis in the pituitary, indicating the mutant mice had insufficient LH secretion. However, the detailed mechanism needs more research.

## Introduction

The *miR-29* family consists of three related mature miRNAs, *miR-29a*, *miR-29b* and *miR-29c*, which are processed from two precursor sequences located at two distinct genomic clusters of *miR-29a/b_1_* and *miR-29b_2_/c*. Members of the miR-29 family are ubiquitously expressed, have considerable overall sequence homology with the same seed sequence. Although they have similar tissue expression patterns, *miR-29a* is the dominant member accounting for more than 50% of total *miR-29* expressed in all tissues (1). *miR-29* play important roles in regulating a number of physiological and pathological processes, including metabolism (1–3), inflammation (4, 5), fibrosis (6), cancer (7) and neurodegeneration (8).

As a potential clinical marker or new form of nucleic acid drug, much attention has been paid to *miR-29* research (9, 10). *miR-29* deficiency causes a wide range of physiological defects in mice. Premature cardiac fibrosis and atherosclerotic plaque remodeling is considered as a result of abnormal expression of *miR-29* target genes *Col4a* (11) and ECM (*Col1a* and *Col5a*) (12), and heart failure and metabolic disorders might be caused by up-regulating the target gene *PCG1α* (1). *miR-29a* responsible for repressing LPL in hepatocytes, contributes to physiological lipid distribution and protects hepatocytes from steatosis (13). Homozygous deletion of *miR-29a/b_1_* in mice led to decreased self-renewal and increased apoptosis in hematopoietic stem cells (HSCs) through up-regulating *Dnmt3a* (14). In addition, early puberty in hypothalamic *miR-29* knockdown females is attributed to ectopic expression of *Tbx21*, a target gene of *miR-29* (15). Reproduction in *miR-29* brain-specifical knockdown mice was affected in a sex-dependent manner, with female mice exhibiting hyperfertility and males being subfertility (16); however, this result is inconsistent with the sterile phenotype reported in the *miR-29a/b_1_* knockout mice (1). Therefore, the relationship between *miR-29a/b_1_* and reproductive function is still not well understood.

In this work, we revealed that female *miR-29a/b_1_* knockout mice exhibit severe fertility problems. We proposed that the lack of *miR-29a/b_1_* in female mice may interfere with the secretion of luteinizing hormone in the pituitary, leading to ovulation failure and a subfertile phenotype.

## Materials and Methods

### Generation of *miR-29a/b_1_* Knockout Mice

A *miR-29a/b_1_* knockout mouse line was established using CRISPR/Cas9 gene editing technology and was supplied by Shanghai Center for Model Organisms (SMOC) (17). *miR-29a/b_1_^−/−^* homozygous animals and their wild-type littermates were obtained by mating corresponding heterozygotes with each other. Genomic DNA was extracted from tail biopsies, using magnetic bead DNA isolation Kit (DE0596D, EmerTher, Shanghai). PCR was adopted for genotyping using 2 × Taq Plus Master Mix (P212-01, Vazyme) under the following conditions: denaturation at 98°C for 2 minutes, then 35 cycles of 98°C for 10 seconds, annealing at 63°C for 15 seconds, and extension at 68°C for 60 seconds. Primers used for genotyping are listed in **Table S1**.

### Animals

All animals were housed in a specific pathogen-free environment (12 h light/12 h dark with lights on at 7.00 h at 21 ± 2°C) with food and water ad libitum. This study was performed in strict accordance with institutional guidelines and approved by the Institutional Animal Care and Use Committee of Shanghai Model Organisms, and the IACUC permit number is 20090002.

### Fertility Assessment

8-week-old *miR-29a/b_1_* KO and wild-type virgin female or male mice were bred with wild-type male or female mice with known fertility at a proportion of ♀2: ♂1, and vaginal plug formation was examined every morning for 20 consecutive days. Pregnant female mice were separated and pups were recorded, while non-pregnant mice continued to mate. Female or male mice that did not conceive within 1 month of mating were defined as infertile.

### Sexual Maturity and Vaginal Smear

Female *miR-29a/b_1_* KO and wild-type mice from the age of 3-week-old were examined twice daily with respect to on vaginal opening as a marker of rodent sexual maturity. The date of vaginal opening in each mouse was recorded. Female *miR-29a/b_1_* KO and wild-type (8-10 weeks old for each genotype) mice were caged individually for 3 weeks and at least two full estrous cycles were obtained in each mouse.

Vaginal smears were collected daily and the determination of estrous cycle was evaluated microscopically with the vaginal epithelium. The vaginal epithelium obtained from the vaginal opening by gently eluting 10 μl of physiological saline solution 2-4 times, then the vaginal epithelium transferred onto a microscopic slide and dried at room temperature and fixed with 100% methanol. The slides were stained with Wright’s Giemsa (BASO) stain and examined with light microscopy. Proestrus cells are well-formed nucleated epithelial cells. Animals with 85% superficial epithelial cells were considered to be estrus. During metestrus, cornified squamous epithelial cells often in fragments, as well as leukocytes, may be observed. Otherwise, the predominant presence of leukocytes in the cytological smear was identified as diestrus.

### Ovariectomy

Adult (8‐10 weeks) *miR-29a/b_1_* KO and control females in diestrus morning were injected subcutaneously with pentobarbital (effective dose 320 mg/kg). Mice were deeply anesthetized and placed on a heating pad. The back skin was shaved and cleaned. About 1.0 cm long incision was made through the muscle layer above the ovaries on each side of the midline. Through the incision, the ovaries were gently pulled outside the body and removed by cauterization below the oviduct. The skin incision was closed with sutures. The mice were left to recover on a heating pad. Adult sham-operated mice were in diestrus on the day of recording as determined by vaginal cytology. Sham‐treated animals were processed in the same way, except for the intact ovaries retained. Mice were killed 7 days post-surgery, and their serum were measured for LH and FSH levels.

### Hormone Measurement

For hormone measurement, orbital blood was collected in the morning (10.00 h-11.00 h) and evening (18.00 h-19.00h) (18) from freely‐moving conscious animals during randomly estrous cycle stages, and were kept at room temperature for 30 minutes. Serum was obtained by centrifuging for 15 minutes at 3000 g at 4°C and was stored at -80°C until analysis. Serum levels of hormone and pituitary proteins LH level were analyzed by Shanghai WESTANG BIO-TECH cooperation using enzyme-linked immunosorbent assay (ELISA). The minimum detectable level of the LH assay was 0.1mIU/ml and the intra-and inter-assay coefficients of variation were 9.9% and 8.3%. The minimum detectable level of the FSH assay was 1mIU/ml and the intra-and inter-assay coefficients of variation were 9.8% and 8.6%, The minimum detectable level of the estrogen assay was 30 pg/ml and the intra-and inter-assay coefficients of variation were 9.3% and 8.5%, The minimum detectable level of the progesterone assay was 0.2ng/ml and the intra-and inter-assay coefficients of variation were 9.5% and 8.3%, The minimum detectable level of the testosterone assay was 0.1ng/ml and the intra-and inter-assay coefficients of variation were 9.4% and 8.2%, respectively.

### GnRH Challenge

Animals received an intraperitoneally injection with 125ng/g (19) exogenous GnRH (L7134, Sigma-Aldrich, St Louis, MO, USA) or saline vehicle. Twenty minutes after GnRH or saline injection, orbital blood was collected, and the resultant serum samples were stored at −80°C for subsequent human-LH radioimmunoassay (RIA, performed by Beijing North Institute Biological Technology, Beijing, China) (20), with sensitivity and intra- and inter-assay coefficient of variation for LH of 0.5 mIU/ml, 15% and 20%, respectively.

### Superovulation and Oocyte Collection

**Superovulation**: To induce superovulation, 8-week-old mice were intraperitoneally injected with 5 IU pregnant mare serum gonadotropin (PMSG, Sigma) at afternoon (15:00h-16:00 h), followed by 5 IU human chorionic gonadotropin (hCG, Sigma) 48 hour later to trigger oocyte maturation and ovulation. Female mice were mated with 10-week-old fertile wild-type males 16 h after injection and checked for vaginal plug formation the next morning.

**Oocyte collection**: Super ovulated or natural mated mice with a visible plug were sacrificed by cervical dislocation, the ovaries were removed and the ampulla was collected. Oocytes were harvested in M2 media and quantified by microscopy (Nikon SMZ800) following brief digestion in hyaluronidase (800IU/ml, Sigma) to strip cumulus and pipetting for 30-60 s. Oocytes were washed 5 times with PBS. The washed oocytes were transferred to M16 media and cultured overnight, and two-cell stage embryos were counted in the next morning.

### LC-MS/MS Analysis

Total pituitary (P) protein from wild-type and *miR-29a/b_1_* KO females (8 weeks, n=3) were isolated and labelled with iTRAQ reagents 114, 115, 116, 117, 118, 119, 120 or 121, respectively, followed by Liquid Chromatography with tandem mass spectrometry (LC-MS/MS) (Shanghai Wayen Biotechnologies Inc.). The mass spectrometry proteomics data have been deposited to the ProteomeXchange Consortium *via* the PRIDE (21) partner repository with the dataset identifier PXD017106.

### Histological Analysis and Follicles Count

Wild-type and *miR-29a/b_1_* KO were euthanized and transcardially perfused with cold saline, followed by 4% paraformaldehyde 0.1 M phosphate buffer (PFA). Brain, pituitary and ovary were collected and fixed overnight at 4°C. Paraffin-embedded ovary samples were serially sectioned at 4 μm-thick sections. Brain coronal (20 μm) slices were cut with a Leica CM1950 following by dehydration in 30% sucrose saline solution.

Pituitary and ovary stained with hematoxylin and eosin using standard histological techniques (Servicebio). Stained sections were scanned using LEICA CTR6000 with a 10X, 20X and 40X objective. Ovarian follicles at different developmental stages were classified and quantified in serial sections according to the Pedersen and Peters method (22). To avoid double counting of follicles across sections, only follicles containing oocyte with a clearly visible nucleus were scored (23), and follicles were counted in every fifth serial section. Any follicle also appearing in the adjacent lookup section was not counted. The entire section was analyzed without subsampling. Each ovary was coded with no information about genotype group for blind counters and prevent bias. The mean count per section was calculated. All follicle types were summed together to determine the total number of follicles.

**For immunohistochemistry**, sections were subjected to antigen retrieval by incubation in 10 mM sodium citrate, pH 7.0, for 10 minutes at 95°C. The endogenous peroxidase activity of the sections was quenched with 3% H_2_O_2_ treatment (Sangon Biotech, Shanghai). Immunohistochemical staining was performed using mouse anti-Lutropin beta antibody (1:500, SANTA CRUZ, sc-373941) or rabbit anti-GnRHR antibody (24, 25) (1:100, Proteintech, 19950-1-AP) and HRP-conjugated donkey anti-mouse IgG (1:1000, ThermoFisher, A16017) or donkey anti-rabbit IgG (1:1000, ThermoFisher, A16035) for lutropin and GnRHR antibody.

**For immunofluorescence**, brain and pituitary sections permeabilized by incubation with 0.1% Triton X-100 in PBS for 10 minutes at room temperature. After permeabilization, the sections were washed three times in PBST, and blocked with 5% normal donkey serum in PBS for 1h at room temperature, then were incubated with mouse anti-Lutropin beta antibody (1:1000, SANTA CRUZ, sc-373941), rabbit anti-GnRH1 (1:500, Immunostar, PA1-121) or rabbit anti-GnRHR antibody (26) (1:100, Proteintech, 19950-1-AP) overnight at 4°C, followed by staining with Alexa Fluor 647-conjugated donkey anti-mouse antibody (Invitrogen Molecular Probes) or Alexa Fluor 594-conjugated donkey anti-rabbit (Invitrogen Molecular Probes) antibody and DAPI dye to stain nuclei. The mouse liver and lung tissues were selected to negative control for GnRHR and Lutropin beta antibody respectively.

Stained sections were scanned using the 40X objective of a Zeiss Confocal microscope (LSM880). The area fractions of positive cells relative to entire area were determined using ImageJ (Fiji, NIH) software. Cell location was mapped to the atlas (27).

### Real-Time Quantitative PCR

Total RNA was isolated using TRIzol (Tiangen Biotech, Beijing) according to the manufacturer’s instructions and kept at -80°C subsequent for use. For microRNA measurement, 2 μg total RNA was transcribed into cDNA using the miRcute Plus miRNA First-Strand cDNA Synthesis Kit (Tiangen Biotech, Beijing). Expression level of mature *miR-29a*, *miR-29b* and *miR-29c* were measured using miRcute Plus miRNA qPCR Detection (Tiangen Biotech, Beijing). *U6* snRNA was used for normalization.

For mRNA measurement, total RNA (2 μg) from each sample was transcribed by using EasyScript First-Strand cDNA Synthesis SuperMix (TransGen Biotech, Beijing), and mRNA levels of target genes were detected using TransStart Tip Green qPCR SuperMix (TransGen Biotech, Beijing) according to the manufacturer’s instructions. Murine *β-actin* was used as a reference to normalize target gene expression levels. Real-time PCR amplification was performed using the Realplex system (Applied Biosystems QuantStudio3, ThermoFisher Scientific). The sequences of the specific primers used are listed in Supplementary Material, **Table S1**. RNA levels were calculated using the 2^−ΔCT^ method, where CT is the cycle threshold (28). Melting curve analysis for each primer set revealed only one peak for each product, and the sizes of PCR products were confirmed by comparing sizes with a commercial ladder after agarose gel electrophoresis. PCR products were further confirmed by sequencing.

### Western Blot

Mice were euthanized and tissues were collected. Total tissue protein was extracted using RIPA buffer (ThermoFisher scientific) containing protease and phosphatase inhibitor cocktails (Selleck Chemicals). Protein concentration was quantified using the Enhanced BCA Protein Assay Kit (Beyotime). Protein (20 μg) from each sample was separated on 4%-20% SDS-PAGE (GenScript) and transferred onto nitrocellulose membranes (GE Healthcare). Membranes were blocked with Western BLoT Blocking Buffer (Protein Free) (Takara) for 1 h at room temperature and then incubated with primary antibodies, Lutropin beta (1:1000, SANTA CRUZ, sc-373941) or anti-β-actin (1:1000, Santa Cruz, sc-47778) diluted in Western BLoT Immuno Booster PF (Takara) at 4°C overnight. After washing with TBST three times, membranes were incubated with fluorescent-conjugated secondary antibody for 1 h (1:10000, LI-COR Biosciences). Quantitative detection of protein expression was then performed using the Odyssey Infrared Imaging system (LI-COR Biosciences) and analyzed with Image J software (National Institutes of Health, Bethesda, MD, USA).

### Statistical Analysis

Data analysis was performed using GraphPad Prism 7 (GraphPad software Inc.). Data are expressed as the mean ± SEM. Difference in mean values between two groups were analyzed using the Student’s t-test (continuous variables) or Mann–Whitney test (discrete variables). For comparisons involving more than two groups, ANOVA (continuous variables) or Kruskal–Wallis (discrete variables) with *post hoc* testing was used, and survival profiles were constructed by Kaplan-Meyer survival analysis. Statistically significant differences are shown with asterisks (*p < 0.05, **p < 0.01, ***p < 0.001, and **** *p* < 0.0001).

## Results

### Genetic Ablation of *miR-29a/b_1_* Leads to Female Sterility

**Figure 1:**
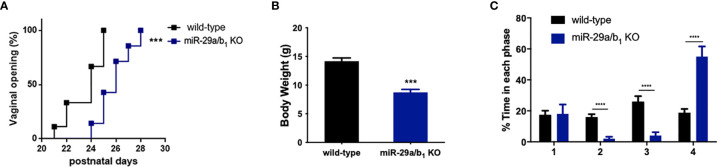
Determination of pubertal onset and estrous cycle in *miR-29a/b_1_* KO females. **(A)** Pubertal onset was determined by vaginal opening in wild-type and *miR-29a/b_1_* KO mice (n=8). **(B)** Body weight of mice at the time of puberty onset (wild-type: 14.18 ± 0.5660, *miR-29a/b_1_* KO: 8.74 ± 0.5202, *p=0.0001*, n=5). **(C)** Estrous cycle quantitative measurements on wild-type and *miR-29a/b_1_*KO females (1: *p=0.9133*, 2-4*: p<0.0001*). ***p < 0.001, ****p < 0.0001.

A conventional *miR-29a/b_1_* knockout mouse line (*miR-29a/b_1_* KO) was previously established using CRISPR/Cas9 methods (17). The genotyping and the expression of *miR-29* in different genotypes of mice were detected by PCR and real-time PCR, respectively (**Figure S1**). To understand the role of *miR-29a/b_1_* in fertility, the reproductive ability of *miR-29a/b_1_* KO mice was evaluated. For data in **Table 1**, of the 25 females tested, 23 were sterile. The two pregnant *miR-29a/b_1_* KO female mice gave birth to two offspring each and were not subsequently pregnant again. Among males, 66.7% *miR-29a/b_1_* KO were still fertile. However, female *miR-29a/b_1_* KO mice exhibited serious reproductive problems. Vaginal plugs were checked to study the mating behavior. *miR-29a/b_1_* KO females had a significant lower mating frequency compared to wild-type females (**Table 1**), suggesting abnormal sexual maturity and estrous cycle. Sexual maturity indicated by vaginal opening occurred 5 days later in *miR-29a/b_1_* KO female mice (postnatal day 28) compared to wild-type littermates (postnatal day 23) (**Figure 1A**). At the time of puberty onset, the mutant mice are significantly lighter than wild-type mice (**Figure 1B**). Meanwhile, abnormal estrous cycle with less time in estrus and metestrus and significantly more time in diestrus was observed in *miR-29a/b_1_* KO female mice. (**Figures 1B, C** and **Figure S2**). RT-PCR analysis revealed that expression of *miR-29a* periodically changed in pituitary and ovarian tissues (**Figure S3A**), suggesting that *miR-29a/b_1_* may play a role in the estrous period in mammals. Taken together, these data illustrate that loss of *miR-29a/b_1_* induces growth retardation in mutant mice and subfertility in females.

**Table 1:** Fertility assessment. Body weight, number of plugs, offspring and pregnancy rate based on mating of wild-type male and female mice.

Fertility assessment	Females		Males	
Genotype	miR-29a/b_1_ KO	Wild-type	miR-29a KO	Wild-type
Body weight (g)	16.35 ± 0.2228	20.72 ± 0.9777	20.02 ± 1.076	23.7 ± 0.6186
Number of plugs	1/25 (4%)	10/12 (83.3)	9/15 (60%)	5/6 (83.3%)
Mean litter size	2	7.6	5.5	7.6
Pregnancies rate (%)	8****	91.7	66.7**	83.3

**p < 0.01, ****p < 0.0001.

### *miR-29a/b_1_* Gene Knockout Leads to Decreased Plasma LH Level and Ovulation Disorder

**Figure 2:**
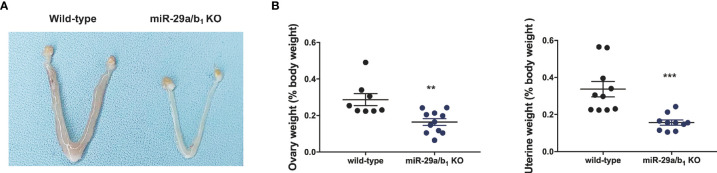
Morphological study of reproductive system. **(A, B)** Macroscopic images, wet ovaries and uteri weight in females, normalized to body weight in the same animals (ovary: wild-type: 0.2868 ± 0.03286, n=8, *miR-29a/b_1_* KO: 0.164 ± 0.01834, n=11, *p=0.0028*; uteri: wild-type: 0.3369 ± 0.04175, *miR-29a/b_1_* KO: 0.1564 ± 0.01394, *p=0.007*, n=10). **p < 0.01, ***p < 0.001.

**Figure 3:**
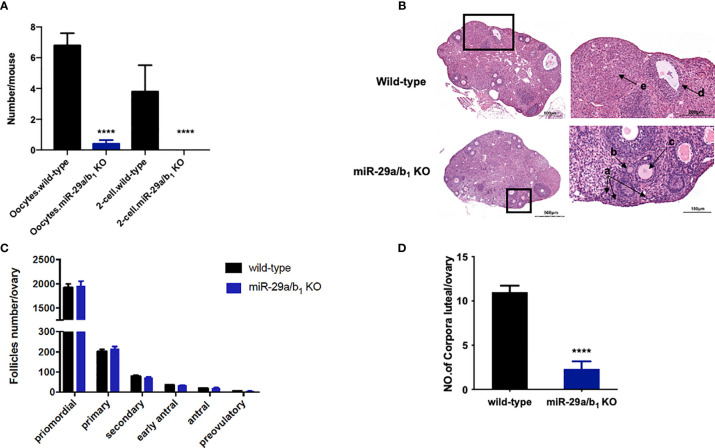
Lacking of *miR-29a/b_1_* impairs ovulation in females. **(A)** Numbers of oocytes and 2-cell embryos in wild-type and *miR-29a/b_1_* KO mice during natural ovulation (n=5). **(B)** Histological sections of ovaries stained with haematoxylin and eosin (H&E) in wild-type and *miR-29a/b_1_* KO mice. Corpora lutea (CLs) and follicles at different stages are shown at higher magnification and denoted with arrows. a: Primordial follicles; b: Primary follicles; c: Secondary follicles; d: Antral follicles; e: Corpora lutea. **(C)** Numbers of follicles at different stages in ovaries from wild-type (n=10) and *miR-29a/b_1_* KO (n=12) mice. Primordial follicles: *p=0.8931*; Primary follicles: *p=0.9802*; Secondary follicles: *p=0.6842*; Early antral follicles: *p=0.5645*; Antral follicles: *p=0.8011*; Preovulatory: *p=0.5081*, respectively. **(D)** Lack of corpora lutea in the ovaries of *miR-29a/b_1_* KO females (*p<0.0001*). ****p < 0.0001.

Ovary and uteri weight in *miR-29a/b_1_* KO females were significantly reduced compared to wild-type females (**Figures 2A, B** **)**, whereas, in males, testis and seminal pouch in *miR-29a/b_1_* KO mice and wild-type counterparts showed no difference (**Figure S4**). Fertilized eggs were collected from the oviducts of wild-type and *miR-29a/b_1_* KO females with vaginal plug after mating with wild-type males. In 20 females *miR-29a/b_1_* KO mice, only 2 oocytes were found and with no two-cell embryos the next day, while among five wild-type mice, 34 oocytes and 19 two-cell embryos were collected (**Figure 3A**). Histomorphometric analysis revealed that mutant ovaries contained normal primordial follicles, a similar number of secondary follicles with normal oocyte and a thick granulosa cell layer, indicating that the early follicles developed normally, but lacked corpora lutea formation (**Figures 3B–D**). These results suggest that subfertility of the mutant female mice may be caused by an ovulation disorder.

**Figure 4:**
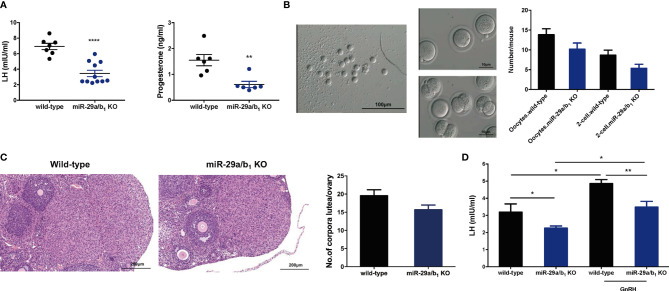
Superovulation rescues the failure in corpora lutea formation in *miR-29a/b_1_* KO mice. **(A)** Serum LH (left) and progesterone (right) levels were significantly reduced in *miR-29a/b_1_* KO compared to wild-type mice (LH: wild-type: 6.927 ± 0.4062 mIU/ml, *miR-29a/b_1_* KO: 3.607 ± 0.5175 mIU/ml, *p*=0.0003, n=7; progesterone: wild-type: 8.166 ± 2.072 nmol/L, n=7, *miR-29a/b_1_* KO: 1.062 ± 0.1181 nmol/L, n=6, *p=0.0092*). **(B)** Numbers of oocytes and 2-cell embryos obtained in response to superovulation in *miR-29a/b_1_* KO and wild-type mice (Oocytes: wild-type: 13.83 ± 1.493, *miR-29a/b_1_* KO: 10.17 ± 1.558, *p=0.1201*; 2-cell embryos: wild-type: 8.667 ± 1.256, *miR-29a/b_1_* KO: 5.333 ± 1.022, *p=0.0666*, n=6). **(C)** Corpora lutea formation in ovaries of *miR-29a/b_1_* KO females after superovulation (wild-type: 19.56 ± 1.634, *miR-29a/b_1_* KO: 15.67 ± 1.302, *p=0.0811*, n=9). **(D)** GnRH challenge in *miR-29a/b_1_* KO and wild-type mice (wild-type: 3.19 ± 0.48 mIU/ml, *miR-29a/b_1_* KO: 2.255 ± 0.1287 mIU/ml, *p=0.0376*, n=5; GnRH: wild-type: 4.857 ± 0.2346 mIU/ml, *p=0.0138*, n=6, *miR-29a/b_1_ KO*: 3.484 ± 0.3357 mIU/ml, *p=0.0145*, n=7). *p < 0.05, **p < 0.01 and ****p < 0.0001.

In females, hormonal control of the estrous cycle and ovulation is essential for the establishment of maturation and fertility in mammals (29). Thus, we examined hormone levels in the serum of wild-type and *miR-29a/b_1_* KO female mice. In the female *miR-29a/b_1_* KO mice, significant decreases in the serum LH and progesterone (P_4_) (**Figure 4A**) were observed, while there was no apparent difference in serum content of follicle-stimulating hormone (FSH) or Testosterone (T) or Estradiol (E_2_) compared to wild-type mice (**Figures S5A–C**). *Cyp19a_1_* and *Cyp17a_1_*, encoding enzymes involved in estradiol and testosterone synthesis, were expressed at identical levels in ovaries from the two groups of mice, while the *Cyp11a*, which essential to the level of sex hormones, was significantly decreased in ovaries from mutant mice (**Figure S5D**). These results indicated that impaired corpora lutea formation in *miR-29a/b_1_* KO mice might be caused by a shortage of LH. This speculation was further confirmed by the superovulation experiment. Ovulation in the mutant mice was rescued by exogenous gonadotropin injection, indicating that responses to LH stimulation were not irreversibly lost in these mutant animals (**Figure 4B**). Ovaries from superovulated adult *miR-29a/b_1_* KO mice showed normal morphology, and the corpora lutea were formed (**Figure 4C**).

**Figure 5:**
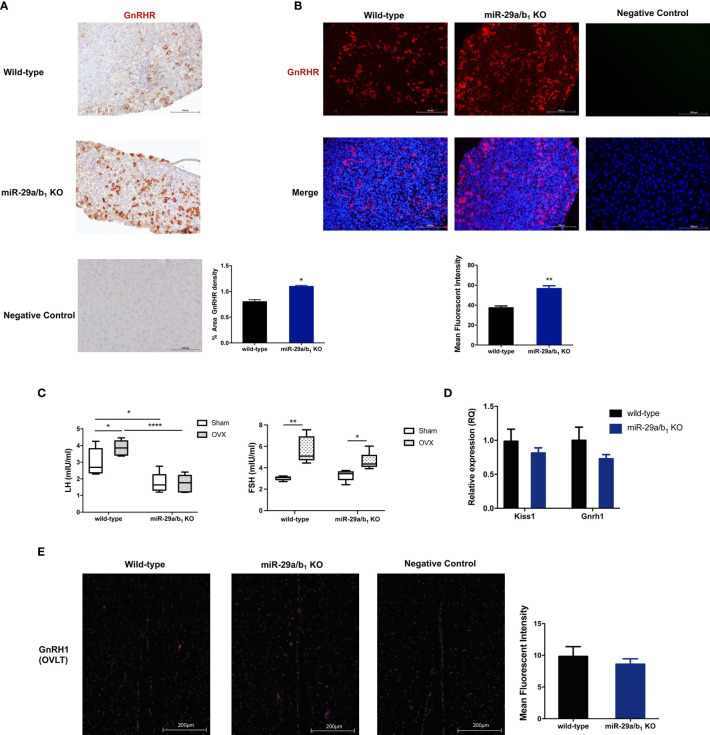
Central mechanism in *miR-29a/b_1_* KO mice. **(A, B)** GnRHR immunoreactivity in pituitary of wild-type and *miR-29a/b_1_* KO females. The receptor was not detectable on the plasma membrane of control. (immunohistochemical: wild-type: 13.43 ± 0.7927, *miR-29a/b_1_* KO: 25.47 ± 0.534, *p=0.0249;* immunofluorescence: wild-type: 37.79 ± 1.858, *miR-29a/b_1_* KO: 56.64 ± 2.767, *p=0.0045*, n=3). **(C)** Serum LH and FSH levels in *miR-29a/b_1_* KO females and controls following ovariectomy (OVX) and sham-operated controls (Sham). (LH: wild-type: *p=0.0401*, *miR-29a/b_1_* KO: *p=0.9249*; FSH: wild-type: *p=0.0016*, *miR-29a/b_1_* KO: *p=0.0185*, n=6). **(D)** Expression of Kiss1 and Gnrh1 in hypothalamus (Gnrh1: wild-type: 1 ± 0.1912, *miR-29a/b_1_* KO: 0.7287 ± 0.06234, *p*=*0.1874*, Kiss1: wild-type: 1 ± 0.1305, *miR-29a/b_1_* KO: 0.8142 ± 0.0757, *p=0.8405*, n=15). **(E)** Normal distribution of GnRH neurons in *miR-29a/b_1_* KO mice compared to control littermates. OVLT, organum vasculosum of the lamina terminalis. Scale bars, 200μm. (wild-type: 9.827 ± 1.547, *miR-29a/b_1_* KO: 8.597 ± 0.8466, *p=0.5238*, n=3). *p < 0.05, **p < 0.01 and ****p < 0.0001.

To determine whether the central regulated mechanisms mediating ovulation were altered in *miR-29a/b_1_* KO mice, females were subsequently treated with an intraperitoneally injection of 125ng/g GnRH or saline vehicle at 10.00 AM. Normal GnRH responsiveness was observed in *miR-29a/b_1_* KO pituitary, but serum LH level in *miR-29a/b_1_* KO females remained markedly below the levels observed in wild-type littermates (**Figure 4D**). Furthermore, The GnRHR-immunoreactivity in the pituitary of *miR-29a/b_1_* KO mice was increased compared to wild-type mice (**Figures 5A, B** **)**, Again, to assess the impact of hyperstimulation with endogenous GnRH modulated by estrogen (30–33), female control and *miR-29a/b_1_* KO animals were castrated or underwent a sham surgery. Animals were euthanized after 7 days, and serum concentrations of LH and FSH were measured. Consistent with control females, castration resulted in an increase in both LH and FSH compared with sham-operated controls, however, the post-castration rise in LH secretion was blocked in *miR-29a/b_1_* KO females, while the FSH level was no significant differences in mutant mice serum from controls (**Figure 5C**). LH levels overall were markable lower in *miR-29a/b_1_* KO females relative to controls. There was no apparent difference in Kiss1and Gnrh1, which stimulating secretion of gonadotropin releasing hormone from the hypothalamus (34–37) and luteinizing hormone from the pituitary (35), respectively (**Figures 5D, E** **)**. These results suggest that ovulation disorder in *miR-29a/b_1_* KO mice might be caused by dysregulation of related pituitary hormones, especially LH.

### Dysregulated Pituitary LHβ Release in *miR-29a/b_1_* KO Mice

**Figure 6:**
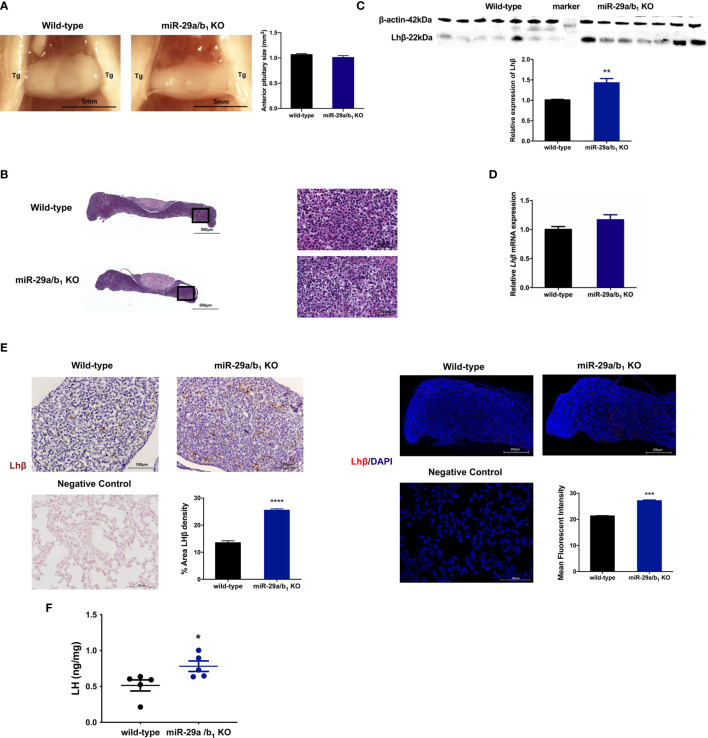
Impairment of Lhβ protein export in the pituitary as a deficiency of *miR-29a/b_1_*. **(A)** Pituitary from female wild-type mice (n=9) and *miR-29a/b_1_* KO mice (n=5) were photographed *in situ* during dissection. Trigeminal nerves that flank the pituitary are marked as Tg. Scale bar = 5 mm (2x magnification). Anterior pituitary size was statistically analyzed (*p=0.2411*, n=7). **(B)** The entire sagittal pituitary and higher magnification in the box from wild-type and *miR-29a/b_1_* KO females are shown. **(C, D)** LHβ protein (*p=0.0019*) and transcripts (*p=0.1278*) levels were determined in pituitary tissues from *miR-29a/b_1_* KO and wild-type mice (n=7). **(E)** Quantification of immunoreactivity LHβ in pituitary of *miR-29a/b_1_* KO or control mice (immunohistochemical: wild-type:13.43 ± 0.7927, n=5, *miR-29a/b_1_* KO: 25.47 ± 0.534, n=4, *p<0.0001*; immunofluorescence: wild-type: 21.27 ± 0.147, *miR-29a/b_1_* KO: 27.11 ± 0.3642, *p=0.0001*, n = 3). LHβ was not detectable on the plasma membrane of control. Scale bars: 200μm. Red indicates positive-LH cells, Cell nuclei (blue) were stained with haematoxylin or DAPI. **(F)** LH proteins relative contents in females. (Wild-type: 0.5147 ± 0.07769, *miR-29a/b_1_* KO: 0.7819 ± 0.07199, *p=0.0357*, n=5). *p < 0.05, **p < 0.01, ***p < 0.001 and ****p < 0.0001.

LH is synthesized in and secreted by the pituitary. A lack of *miR-29a/b_1_* was confirmed in mutant pituitary tissues (**Figure S3B**). The anterior pituitary undergoes rapid proliferation in neonatal mice, subsequently expanding the cells that produce factors required for growth and reproduction (38). Defective anterior pituitary development in animals contributes to many organism-level developmental defects (39). However, there was no difference in pituitary structure, size or position of the anterior pituitary between wild-type and *miR-29a/b_1_* KO mice (**Figure 6A**). No abnormalities were found upon pathological examination of mutant pituitary tissues (**Figure 6B**). Notably, transcript levels of the *Lhβ* gene in *miR-29a/b_1_* KO pituitary did not differ from control animals, but LH protein level and immunoreactivity were even higher in KO mice (**Figures 6C–F**).

**Figure 7:**
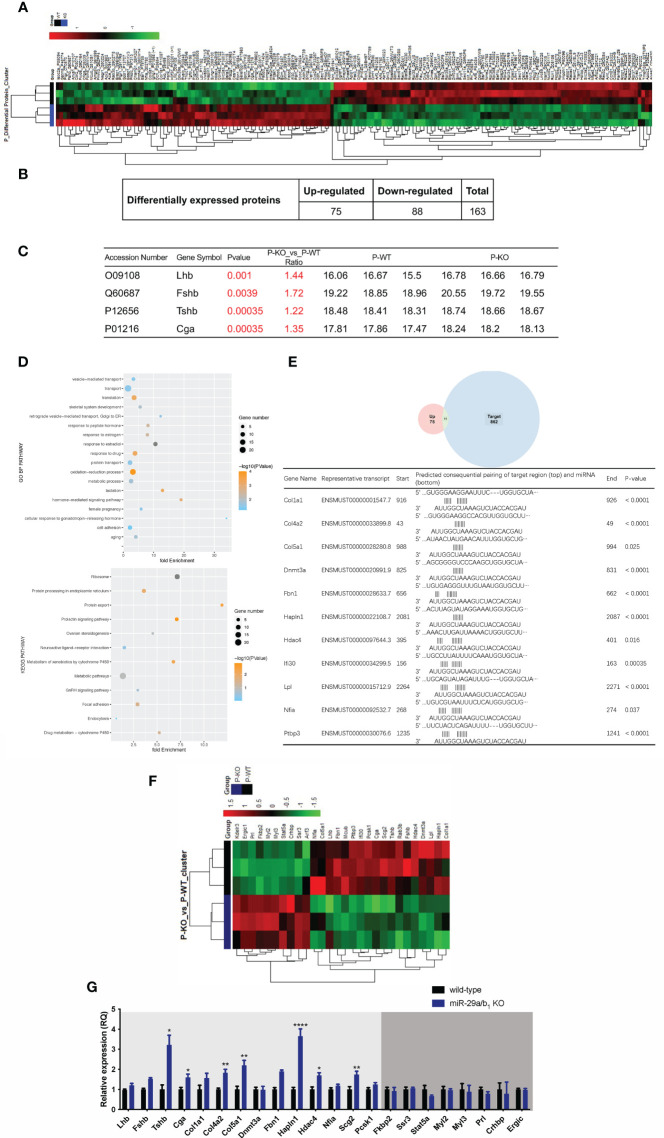
Comparing protein expression profile in the pituitary of wild-type and *miR-29a/b_1_* KO mice. **(A, B)** Differential protein from pituitary of *miR-29a/b_1_* KO and wild-type mice (n=3 for each) detected by MS. **(C)** Pituitary hormone expression. **(D)** GO and KEGG analysis of the pituitary from *miR-29a/b_1_* KO compared to wild-type mice. **(E)** 11predicted miR-29a targets from up-regulated proteins. **(F)** Heat map of genes about vesicle-transport. **(G)** Quantification of up-regulation genes including coded pituitary hormone (light gray shaded area) and down-regulation vesicle-transport activators (dark gray shaded area) (Lhb: *p=0.2411, Fshb: p=0.0002, Tshb: p=0.0017, Cga: p=0.0117, Col1a1: p=0.1038, Col4a2: p=0.0012, Col5a1: p=0.0023, Dnmt3a: p=0.9237, Fbn1: p<0.0001, Hpln1: p<0.0001, Hdac4: p=0.0332, Nfia: p=0.1379, Scg2: p=0.0058, Pcsk1: p=0.0743, Fkbp2: p=0.6975, Ssr3: p=0.6844, Stat5a:0.1329, Myl2: p=0.7879, Myl3: p=0.7858, Prl: p=0.1706, Crhbp: p=0.7510, Ergic: p=0.7937*, n=6). *p < 0.05, **p < 0.01 and ****p < 0.0001.

To further elucidate the effects of *miR-29a/b_1_* gene knockout on pituitary function, iTRAQ analysis was performed to compare proteomic changes in the pituitary between mutant and wild-type mice. Total pituitary protein from three biological replicates of each genotype were subjected to LC-MS/MS analysis. The hierarchical clustering profile of differential proteins is shown in the heat map (**Figure 7A**). A total of 163 cellular proteins were statistically significant altered (*p<0.05*), including 75 upregulated proteins and 88 downregulated proteins (**Figure 7B**, **Table 2**). LHβ and FSHβ were significantly increased in the pituitary of *miR-29a/b_1_* KO mice according to b/y ion signal intensity (**Figure 7C**). Besides, TSHβ and Cga were also markedly upregulated as a result of the *miR-29a/b_1_*deficiency (**Figures 7C, G** **)**. Through GO analysis, altered proteins identified in this study were found to be involved in a wide range of biological process, and most of the differential proteins were classified in the protein transport processes, which are essential for vesicle-mediated transport in the cytoplasm and exocytosis during plasma infusion (40–42) (**Figure 7D**).

**Table 2:** Differentially expressed proteins in pituitary involved in miR-29 regulation and protein transport (*p<0.05 and fold change≥1.2 or ≤ 0.83*).

Accession Number	Gene Symbol	Identified Proteins	Molecular Weight	P value	Ratio (KO vs wild-type)
Q60687	Fshb	Follitropin subunit beta OS=Mus musculus GN=Fshb PE=2 SV=1	15 kDa	0.0039	1.72
P32848	Pvalb	Parvalbumin alpha OS=Mus musculus GN=Pvalb PE=1 SV=3	12 kDa	0.0065	1.65
Q9Z0F7	Sncg	Gamma-synuclein OS=Mus musculus GN=Sncg PE=1 SV=1	13 kDa	< 0.0001	1.55
P01887	B2m	Beta-2-microglobulin OS=Mus musculus GN=B2m PE=1 SV=2	14 kDa	0.0039	1.54
Q80TB8	Vat1l	Synaptic vesicle membrane protein VAT-1 homolog-like OS=Mus musculus GN=Vat1l PE=1 SV=2	46 kDa	< 0.0001	1.48
Q03517	Scg2	Secretogranin-2 OS=Mus musculus GN=Scg2 PE=1 SV=1	71 kDa	< 0.0001	1.47
O09108	Lhb	Lutropin subunit beta OS=Mus musculus GN=Lhb PE=2 SV=2	15 kDa	0.001	1.44
Q9CYK2	Qpct	Glutaminyl-peptide cyclotransferase OS=Mus musculus GN=Qpct PE=1 SV=2	41 kDa	< 0.0001	1.44
Q9ESY9	Ifi30	Gamma-interferon-inducible lysosomal thiol reductase OS=Mus musculus GN=Ifi30 PE=1 SV=3	28 kDa	0.00035	1.41
Q60963	Pla2g7	Platelet-activating factor acetylhydrolase OS=Mus musculus GN=Pla2g7 PE=2 SV=2	49 kDa	< 0.0001	1.40
O70570	Pigr	Polymeric immunoglobulin receptor OS=Mus musculus GN=Pigr PE=1 SV=1	85 kDa	0.037	1.38
P33267	Cyp2f2	Cytochrome P450 2F2 OS=Mus musculus GN=Cyp2f2 PE=1 SV=1	56 kDa	< 0.0001	1.37
Q8R3N6	Thoc1	THO complex subunit 1 OS=Mus musculus GN=Thoc1 PE=1 SV=1	75 kDa	0.025	1.37
P01216	Cga	Glycoprotein hormones alpha chain OS=Mus musculus GN=Cga PE=2 SV=1	14 kDa	0.00035	1.35
P32037	Slc2a3	Solute carrier family 2, facilitated glucose transporter member 3 OS=Mus musculus GN=Slc2a3 PE=1 SV=1	53 kDa	< 0.0001	1.35
Q8VCT4	Ces1d	Carboxylesterase 1D OS=Mus musculus GN=Ces1d PE=1 SV=1	62 kDa	< 0.0001	1.34
P30115	Gsta3	Glutathione S-transferase A3 OS=Mus musculus GN=Gsta3 PE=1 SV=2	25 kDa	0.0039	1.34
**P08122**	**Col4a2**	**Collagen alpha-2(IV) chain OS=Mus musculus GN=Col4a2 PE=1 SV=4**	**167 kDa**	**< 0.0001**	**1.33**
**P52927**	**Hmga2**	**High mobility group protein HMGI-C OS=Mus musculus GN=Hmga2 PE=1 SV=1**	**12 kDa**	**0.00035**	**1.33**
O55100	Syngr1	Synaptogyrin-1 OS=Mus musculus GN=Syngr1 PE=1 SV=2	26 kDa	0.0039	1.33
Q64524	Hist2h2be	Histone H2B type 2-E OS=Mus musculus GN=Hist2h2be PE=1 SV=3	14 kDa	0.0039	1.32
Q9QXF8	Gnmt	Glycine N-methyltransferase OS=Mus musculus GN=Gnmt PE=1 SV=3	33 kDa	0.0039	1.32
Q8VDW0	Ddx39a	ATP-dependent RNA helicase DDX39A OS=Mus musculus GN=Ddx39a PE=1 SV=1	49 kDa	0.016	1.32
A9Z1V5	Vwa5b1	von Willebrand factor A domain-containing protein 5B1 OS=Mus musculus GN=Vwa5b1 PE=2 SV=1	134 kDa	0.0039	1.32
**Q6NZM9**	**Hdac4**	**Histone deacetylase 4 OS=Mus musculus GN=Hdac4 PE=1 SV=1**	**119 kDa**	**0.016**	**1.32**
G3X982	Aox3	Aldehyde oxidase 3 OS=Mus musculus GN=Aox3 PE=1 SV=1	147 kDa	0.0065	1.32
P47739	Aldh3a1	Aldehyde dehydrogenase, dimeric NADP-preferring OS=Mus musculus GN=Aldh3a1 PE=1 SV=2	50 kDa	< 0.0001	1.31
Q9D164	Fxyd6	FXYD domain-containing ion transport regulator 6 OS=Mus musculus GN=Fxyd6 PE=1 SV=2	10 kDa	0.0065	1.31
Q9EQH2	Erap1	Endoplasmic reticulum aminopeptidase 1 OS=Mus musculus GN=Erap1 PE=1 SV=2	107 kDa	< 0.0001	1.30
P01868 (+1)	Ighg1	Ig gamma-1 chain C region secreted form OS=Mus musculus GN=Ighg1 PE=1 SV=1	36 kDa	0.00035	1.30
Q9QUP5	Hapln1	Hyaluronan and proteoglycan link protein 1 OS=Mus musculus GN=Hapln1 PE=1 SV=1	40 kDa	< 0.0001	1.29
**O88508**	**Dnmt3a**	**DNA (cytosine-5)-methyltransferase 3A OS=Mus musculus GN=Dnmt3a PE=1 SV=2**	**102 kDa**	**< 0.0001**	**1.29**
Q07079	Igfbp5	Insulin-like growth factor-binding protein 5 OS=Mus musculus GN=Igfbp5 PE=1 SV=1	30 kDa	< 0.0001	1.28
P26339	Chga	Chromogranin-A OS=Mus musculus GN=Chga PE=1 SV=1	52 kDa	< 0.0001	1.27
P09602	Hmgn2	Non-histone chromosomal protein HMG-17 OS=Mus musculus GN=Hmgn2 PE=1 SV=2	9 kDa	< 0.0001	1.27
Q9CZT8	Rab3b	Ras-related protein Rab-3B OS=Mus musculus GN=Rab3b PE=1 SV=1	25 kDa	< 0.0001	1.27
P28654	Dcn	Decorin OS=Mus musculus GN=Dcn PE=1 SV=1	40 kDa	< 0.0001	1.27
P85094	Isoc2a	Isochorismatase domain-containing protein 2A OS=Mus musculus GN=Isoc2a PE=1 SV=1	22 kDa	0.0027	1.27
P22005	Penk	Proenkephalin-A OS=Mus musculus GN=Penk PE=1 SV=2	31 kDa	0.01	1.27
P02301 (+1)	H3f3c	Histone H3.3C OS=Mus musculus GN=H3f3c PE=3 SV=3	15 kDa	0.0039	1.27
Q91XV3	Basp1	Brain acid soluble protein 1 OS=Mus musculus GN=Basp1 PE=1 SV=3	22 kDa	< 0.0001	1.27
**Q02780**	**Nfia**	**Nuclear factor 1 A-type OS=Mus musculus GN=Nfia PE=1 SV=1**	**59 kDa**	**0.037**	**1.27**
Q8K327	Champ1	Chromosome alignment-maintaining phosphoprotein 1 OS=Mus musculus GN=Champ1 PE=1 SV=1	88 kDa	0.0017	1.27
**Q6ZPF4**	**Fmnl3**	**Formin-like protein 3 OS=Mus musculus GN=Fmnl3 PE=1 SV=2**	**117 kDa**	**0.037**	**1.27**
P82198	Tgfbi	Transforming growth factor-beta-induced protein ig-h3 OS=Mus musculus GN=Tgfbi PE=1 SV=1	75 kDa	< 0.0001	1.26
Q61599	Arhgdib	Rho GDP-dissociation inhibitor 2 OS=Mus musculus GN=Arhgdib PE=1 SV=3	23 kDa	0.00034	1.26
Q80W14	Prpf40b	Pre-mRNA-processing factor 40 homolog B OS=Mus musculus GN=Prpf40b PE=1 SV=2	99 kDa	0.0039	1.26
P97467	Pam	Peptidyl-glycine alpha-amidating monooxygenase OS=Mus musculus GN=Pam PE=1 SV=2	109 kDa	< 0.0001	1.25
**P11152**	**Lpl**	**Lipoprotein lipase OS=Mus musculus GN=Lpl PE=1 SV=3**	**53 kDa**	**< 0.0001**	**1.25**
Q00519	Xdh	Xanthine dehydrogenase/oxidase OS=Mus musculus GN=Xdh PE=1 SV=5	147 kDa	0.024	1.25
Q91YR9	Ptgr1	Prostaglandin reductase 1 OS=Mus musculus GN=Ptgr1 PE=1 SV=2	36 kDa	0.0013	1.25
**P11087**	**Col1a1**	**Collagen alpha-1(I) chain OS=Mus musculus GN=Col1a1 PE=1 SV=4**	**138 kDa**	**< 0.0001**	**1.24**
P10107	Anxa1	Annexin A1 OS=Mus musculus GN=Anxa1 PE=1 SV=2	39 kDa	< 0.0001	1.24
P13707	Gpd1	Glycerol-3-phosphate dehydrogenase [NAD(+)], cytoplasmic OS=Mus musculus GN=Gpd1 PE=1 SV=3	38 kDa	< 0.0001	1.24
**O88207**	**Col5a1**	**Collagen alpha-1(V) chain OS=Mus musculus GN=Col5a1 PE=1 SV=2**	**184 kDa**	**0.025**	**1.24**
**Q61554**	**Fbn1**	**Fibrillin-1 OS=Mus musculus GN=Fbn1 PE=1 SV=2**	**312 kDa**	**< 0.0001**	**1.23**
Q05816	Fabp5	Fatty acid-binding protein, epidermal OS=Mus musculus GN=Fabp5 PE=1 SV=3	15 kDa	< 0.0001	1.23
P08074	Cbr2	Carbonyl reductase [NADPH] 2 OS=Mus musculus GN=Cbr2 PE=1 SV=1	26 kDa	0.0013	1.23
P97313	Prkdc	DNA-dependent protein kinase catalytic subunit OS=Mus musculus GN=Prkdc PE=1 SV=3	471 kDa	0.031	1.23
**P19785**	**Esr1**	**Estrogen receptor OS=Mus musculus GN=Esr1 PE=1 SV=1**	**67 kDa**	**0.0031**	**1.23**
P63239	Pcsk1	Neuroendocrine convertase 1 OS=Mus musculus GN=Pcsk1 PE=1 SV=1	84 kDa	< 0.0001	1.22
**Q8BHD7**	**Ptbp3**	**Polypyrimidine tract-binding protein 3 OS=Mus musculus GN=Ptbp3 PE=1 SV=1**	**57 kDa**	**< 0.0001**	**1.22**
Q9WUB3	Pygm	Glycogen phosphorylase, muscle form OS=Mus musculus GN=Pygm PE=1 SV=3	97 kDa	< 0.0001	1.22
P12656	Tshb	Thyrotropin subunit beta OS=Mus musculus GN=Tshb PE=2 SV=1	15 kDa	0.00035	1.22
Q9WVH9	Fbln5	Fibulin-5 OS=Mus musculus GN=Fbln5 PE=1 SV=1	50 kDa	0.016	1.22
P35455	Avp	Vasopressin-neurophysin 2-copeptin OS=Mus musculus GN=Avp PE=2 SV=1	18 kDa	< 0.0001	1.21
O70624	Myoc	Myocilin OS=Mus musculus GN=Myoc PE=1 SV=1	55 kDa	< 0.0001	1.21
P09470	Ace	Angiotensin-converting enzyme OS=Mus musculus GN=Ace PE=1 SV=3	151 kDa	0.0006	1.21
P11404	Fabp3	Fatty acid-binding protein, heart OS=Mus musculus GN=Fabp3 PE=1 SV=5	15 kDa	< 0.0001	1.21
Q80Z24	Negr1	Neuronal growth regulator 1 OS=Mus musculus GN=Negr1 PE=1 SV=1	38 kDa	0.01	1.21
P47738	Aldh2	Aldehyde dehydrogenase, mitochondrial OS=Mus musculus GN=Aldh2 PE=1 SV=1	57 kDa	< 0.0001	1.21
P17563	Selenbp1	Selenium-binding protein 1 OS=Mus musculus GN=Selenbp1 PE=1 SV=2	53 kDa	< 0.0001	1.21
P48774	Gstm5	Glutathione S-transferase Mu 5 OS=Mus musculus GN=Gstm5 PE=1 SV=1	27 kDa	< 0.0001	1.21
Q8R0F9	Sec14l4	SEC14-like protein 4 OS=Mus musculus GN=Sec14l4 PE=1 SV=1	46 kDa	< 0.0001	1.21
Q810S1	Mcub	Calcium uniporter regulatory subunit MCUb, mitochondrial OS=Mus musculus GN=Mcub PE=1 SV=1	40 kDa	0.00034	1.21
P81117	Nucb2	Nucleobindin-2 OS=Mus musculus GN=Nucb2 PE=1 SV=2	50 kDa	< 0.0001	0.83
P62852	Rps25	40S ribosomal protein S25 OS=Mus musculus GN=Rps25 PE=1 SV=1	14 kDa	< 0.0001	0.83
P84084	Arf5	ADP-ribosylation factor 5 OS=Mus musculus GN=Arf5 PE=1 SV=2	21 kDa	< 0.0001	0.83
P10852	Slc3a2	4F2 cell-surface antigen heavy chain OS=Mus musculus GN=Slc3a2 PE=1 SV=1	58 kDa	< 0.0001	0.83
Q9DC16	Ergic1	Endoplasmic reticulum-Golgi intermediate compartment protein 1 OS=Mus musculus GN=Ergic1 PE=1 SV=1	33 kDa	< 0.0001	0.83
Q9JJI8	Rpl38	60S ribosomal protein L38 OS=Mus musculus GN=Rpl38 PE=1 SV=3	8 kDa	< 0.0001	0.83
P50096	Impdh1	Inosine-5'-monophosphate dehydrogenase 1 OS=Mus musculus GN=Impdh1 PE=1 SV=2	55 kDa	< 0.0001	0.83
Q9QYI6	Dnajb9	DnaJ homolog subfamily B member 9 OS=Mus musculus GN=Dnajb9 PE=1 SV=2	26 kDa	0.0027	0.83
Q91V04	Tram1	Translocating chain-associated membrane protein 1 OS=Mus musculus GN=Tram1 PE=1 SV=3	43 kDa	< 0.0001	0.83
Q9JHH9	Copz2	Coatomer subunit zeta-2 OS=Mus musculus GN=Copz2 PE=1 SV=1	23 kDa	0.00093	0.83
P25322	Ccnd1	G1/S-specific cyclin-D1 OS=Mus musculus GN=Ccnd1 PE=1 SV=1	33 kDa	0.00049	0.83
Q922H9	Znf330	Zinc finger protein 330 OS=Mus musculus GN=Znf330 PE=1 SV=1	36 kDa	0.00035	0.83
Q80UM7	Mogs	Mannosyl-oligosaccharide glucosidase OS=Mus musculus GN=Mogs PE=1 SV=1	92 kDa	< 0.0001	0.82
Q99KK2	Cmas	N-acylneuraminate cytidylyltransferase OS=Mus musculus GN=Cmas PE=1 SV=2	48 kDa	< 0.0001	0.82
Q5I012	Slc38a10	Putative sodium-coupled neutral amino acid transporter 10 OS=Mus musculus GN=Slc38a10 PE=1 SV=2	117 kDa	< 0.0001	0.82
P62267	Rps23	40S ribosomal protein S23 OS=Mus musculus GN=Rps23 PE=1 SV=3	16 kDa	< 0.0001	0.82
P83882	Rpl36a	60S ribosomal protein L36a OS=Mus musculus GN=Rpl36a PE=1 SV=2	12 kDa	< 0.0001	0.82
P60867	Rps20	40S ribosomal protein S20 OS=Mus musculus GN=Rps20 PE=1 SV=1	13 kDa	< 0.0001	0.82
Q9D823	Rpl37	60S ribosomal protein L37 OS=Mus musculus GN=Rpl37 PE=3 SV=3	11 kDa	< 0.0001	0.82
Q3TJZ6	Fam98a	Protein FAM98A OS=Mus musculus GN=Fam98a PE=1 SV=1	55 kDa	0.00012	0.82
Q8K221	Arfip2	Arfaptin-2 OS=Mus musculus GN=Arfip2 PE=1 SV=2	38 kDa	0.00035	0.82
P62862	Fau	40S ribosomal protein S30 OS=Mus musculus GN=Fau PE=1 SV=1	7 kDa	0.00035	0.82
Q9Z0S9	Rabac1	Prenylated Rab acceptor protein 1 OS=Mus musculus GN=Rabac1 PE=1 SV=1	21 kDa	0.0039	0.82
B9EJR8	Dnaaf5	Dynein assembly factor 5, axonemal OS=Mus musculus GN=Dnaaf5 PE=1 SV=1	94 kDa	0.047	0.82
Q9CZB0	Sdhc	Succinate dehydrogenase cytochrome b560 subunit, mitochondrial OS=Mus musculus GN=Sdhc PE=1 SV=1	18 kDa	0.016	0.82
Q8VDJ3	Hdlbp	Vigilin OS=Mus musculus GN=Hdlbp PE=1 SV=1	142 kDa	< 0.0001	0.82
Q8BP67	Rpl24	60S ribosomal protein L24 OS=Mus musculus GN=Rpl24 PE=1 SV=2	18 kDa	< 0.0001	0.82
Q9D1R9	Rpl34	60S ribosomal protein L34 OS=Mus musculus GN=Rpl34 PE=1 SV=2	13 kDa	< 0.0001	0.82
P45878	Fkbp2	Peptidyl-prolyl cis-trans isomerase FKBP2 OS=Mus musculus GN=Fkbp2 PE=1 SV=1	15 kDa	< 0.0001	0.82
P60202	Plp1	Myelin proteolipid protein OS=Mus musculus GN=Plp1 PE=1 SV=2	30 kDa	< 0.0001	0.82
P33622	Apoc3	Apolipoprotein C-III OS=Mus musculus GN=Apoc3 PE=1 SV=2	11 kDa	0.021	0.82
Q9DCF9	Ssr3	Translocon-associated protein subunit gamma OS=Mus musculus GN=Ssr3 PE=1 SV=1	21 kDa	< 0.0001	0.82
Q03157	Aplp1	Amyloid-like protein 1 OS=Mus musculus GN=Aplp1 PE=1 SV=1	73 kDa	0.019	0.81
Q8CI11	Gnl3	Guanine nucleotide-binding protein-like 3 OS=Mus musculus GN=Gnl3 PE=1 SV=2	61 kDa	< 0.0001	0.81
Q4PJX1	Odr4	Protein odr-4 homolog OS=Mus musculus GN=Odr4 PE=1 SV=2	50 kDa	0.00067	0.81
Q91XC8	Dap	Death-associated protein 1 OS=Mus musculus GN=Dap PE=1 SV=3	11 kDa	0.0065	0.81
Q01768	Nme2	Nucleoside diphosphate kinase B OS=Mus musculus GN=Nme2 PE=1 SV=1	17 kDa	< 0.0001	0.81
C0HK80	Arxes2	Adipocyte-related X-chromosome expressed sequence 2 OS=Mus musculus GN=Arxes2 PE=1 SV=1	20 kDa	0.00022	0.81
Q80WW9	Ddrgk1	DDRGK domain-containing protein 1 OS=Mus musculus GN=Ddrgk1 PE=1 SV=2	36 kDa	0.0032	0.81
P42230	Stat5a	Signal transducer and activator of transcription 5A OS=Mus musculus GN=Stat5a PE=1 SV=1	91 kDa	< 0.0001	0.81
Q3TMP8	Tmem38a	Trimeric intracellular cation channel type A OS=Mus musculus GN=Tmem38a PE=1 SV=2	33 kDa	0.031	0.81
Q922Q8	Lrrc59	Leucine-rich repeat-containing protein 59 OS=Mus musculus GN=Lrrc59 PE=1 SV=1	35 kDa	< 0.0001	0.80
O55142	Rpl35a	60S ribosomal protein L35a OS=Mus musculus GN=Rpl35a PE=1 SV=2	13 kDa	< 0.0001	0.80
P61961	Ufm1	Ubiquitin-fold modifier 1 OS=Mus musculus GN=Ufm1 PE=1 SV=1	9 kDa	0.00017	0.80
P47964	Rpl36	60S ribosomal protein L36 OS=Mus musculus GN=Rpl36 PE=3 SV=2	12 kDa	0.0039	0.80
Q99PL5	Rrbp1	Ribosome-binding protein 1 OS=Mus musculus GN=Rrbp1 PE=1 SV=2	173 kDa	< 0.0001	0.80
Q9R0P6	Sec11a	Signal peptidase complex catalytic subunit SEC11A OS=Mus musculus GN=Sec11a PE=1 SV=1	21 kDa	< 0.0001	0.80
Q9CY50	Ssr1	Translocon-associated protein subunit alpha OS=Mus musculus GN=Ssr1 PE=1 SV=1	32 kDa	0.0012	0.80
Q9D8S4	Rexo2	Oligoribonuclease, mitochondrial OS=Mus musculus GN=Rexo2 PE=1 SV=2	27 kDa	< 0.0001	0.80
Q8R1L4	Kdelr3	ER lumen protein-retaining receptor 3 OS=Mus musculus GN=Kdelr3 PE=1 SV=1	25 kDa	0.00035	0.80
P47199	Cryz	Quinone oxidoreductase OS=Mus musculus GN=Cryz PE=1 SV=1	35 kDa	< 0.0001	0.79
Q64674	Srm	Spermidine synthase OS=Mus musculus GN=Srm PE=1 SV=1	34 kDa	< 0.0001	0.79
Q8K009	Aldh1l2	Mitochondrial 10-formyltetrahydrofolate dehydrogenase OS=Mus musculus GN=Aldh1l2 PE=1 SV=2	102 kDa	< 0.0001	0.79
Q8R1U2	Cgref1	Cell growth regulator with EF hand domain protein 1 OS=Mus musculus GN=Cgref1 PE=1 SV=1	31 kDa	0.00015	0.79
Q8VEL9	Rem2	GTP-binding protein REM 2 OS=Mus musculus GN=Rem2 PE=1 SV=2	37 kDa	< 0.0001	0.79
P21956	Mfge8	Lactadherin OS=Mus musculus GN=Mfge8 PE=1 SV=3	51 kDa	< 0.0001	0.78
Q9D8V7	Sec11c	Signal peptidase complex catalytic subunit SEC11C OS=Mus musculus GN=Sec11c PE=1 SV=3	22 kDa	< 0.0001	0.78
Q9CXI5	Manf	Mesencephalic astrocyte-derived neurotrophic factor OS=Mus musculus GN=Manf PE=1 SV=1	20 kDa	< 0.0001	0.78
O70251	Eef1b	Elongation factor 1-beta OS=Mus musculus GN=Eef1b PE=1 SV=5	25 kDa	< 0.0001	0.77
Q78XF5	Ostc	Oligosaccharyltransferase complex subunit OSTC OS=Mus musculus GN=Ostc PE=1 SV=1	17 kDa	0.0039	0.77
Q9CQS8	Sec61b	Protein transport protein Sec61 subunit beta OS=Mus musculus GN=Sec61b PE=1 SV=3	10 kDa	< 0.0001	0.77
Q61036	Pak3	Serine/threonine-protein kinase PAK 3 OS=Mus musculus GN=Pak3 PE=1 SV=2	62 kDa	< 0.0001	0.77
Q91X91	Qprt	Nicotinate-nucleotide pyrophosphorylase [carboxylating] OS=Mus musculus GN=Qprt PE=1 SV=1	32 kDa	0.0031	0.77
Q61941	Nnt	NAD(P) transhydrogenase, mitochondrial OS=Mus musculus GN=Nnt PE=1 SV=2	114 kDa	< 0.0001	0.76
Q05186	Rcn1	Reticulocalbin-1 OS=Mus musculus GN=Rcn1 PE=1 SV=1	38 kDa	< 0.0001	0.76
C0HKG5	Rnaset2a	Ribonuclease T2-A OS=Mus musculus GN=Rnaset2a PE=1 SV=1	30 kDa	< 0.0001	0.76
Q8K023	Akr1c18	Aldo-keto reductase family 1 member C18 OS=Mus musculus GN=Akr1c18 PE=1 SV=2	37 kDa	0.0039	0.76
Q8R059	Gale	UDP-glucose 4-epimerase OS=Mus musculus GN=Gale PE=1 SV=1	38 kDa	< 0.0001	0.76
P61205	Arf3	ADP-ribosylation factor 3 OS=Mus musculus GN=Arf3 PE=2 SV=2	21 kDa	< 0.0001	0.75
P23927	Cryab	Alpha-crystallin B chain OS=Mus musculus GN=Cryab PE=1 SV=2	20 kDa	0.0039	0.75
Q8BH97	Rcn3	Reticulocalbin-3 OS=Mus musculus GN=Rcn3 PE=1 SV=1	38 kDa	< 0.0001	0.74
Q8CFA2	Amt	Aminomethyltransferase, mitochondrial OS=Mus musculus GN=Amt PE=1 SV=1	44 kDa	< 0.0001	0.74
Q922W5	Pycr1	Pyrroline-5-carboxylate reductase 1, mitochondrial OS=Mus musculus GN=Pycr1 PE=1 SV=1	32 kDa	< 0.0001	0.74
P34884	Mif	Macrophage migration inhibitory factor OS=Mus musculus GN=Mif PE=1 SV=2	13 kDa	< 0.0001	0.73
Q9D7S7	Rpl22l1	60S ribosomal protein L22-like 1 OS=Mus musculus GN=Rpl22l1 PE=1 SV=1	14 kDa	0.00049	0.73
Q9WUT3	Rps6ka2	Ribosomal protein S6 kinase alpha-2 OS=Mus musculus GN=Rps6ka2 PE=1 SV=1	83 kDa	< 0.0001	0.69
Q9D1M7	Fkbp11	Peptidyl-prolyl cis-trans isomerase FKBP11 OS=Mus musculus GN=Fkbp11 PE=1 SV=1	22 kDa	< 0.0001	0.69
P07759	Serpina3k	Serine protease inhibitor A3K OS=Mus musculus GN=Serpina3k PE=1 SV=2	47 kDa	< 0.0001	0.69
Q60841	Reln	Reelin OS=Mus musculus GN=Reln PE=1 SV=3	387 kDa	< 0.0001	0.66
P61750	Arf4	ADP-ribosylation factor 4 OS=Mus musculus GN=Arf4 PE=1 SV=2	20 kDa	0.00035	0.66
Q60590	Orm1	Alpha-1-acid glycoprotein 1 OS=Mus musculus GN=Orm1 PE=1 SV=1	24 kDa	0.00035	0.66
P47212	Gal	Galanin peptides OS=Mus musculus GN=Gal PE=2 SV=1	13 kDa	0.0039	0.65
P51667	Myl2	Myosin regulatory light chain 2, ventricular/cardiac muscle isoform OS=Mus musculus GN=Myl2 PE=1 SV=3	19 kDa	< 0.0001	0.64
P06879	Prl	Prolactin OS=Mus musculus GN=Prl PE=2 SV=1	25 kDa	< 0.0001	0.61
Q640N1	Aebp1	Adipocyte enhancer-binding protein 1 OS=Mus musculus GN=Aebp1 PE=1 SV=1	128 kDa	< 0.0001	0.57
Q60571	Crhbp	Corticotropin-releasing factor-binding protein OS=Mus musculus GN=Crhbp PE=2 SV=1	36 kDa	0.00035	0.53
P09542	Myl3	Myosin light chain 3 OS=Mus musculus GN=Myl3 PE=1 SV=4	22 kDa	0.00035	0.46

The intersected gene between upregulated expression and miR-29a targets through miRDB (http://mirdb.org) were analyzed, 11 potential direct target transcripts of miR-29a were discovered (**Figure 7E**), and predicted target genes were expected to be upregulated in miRNA loss of-function models (**Figure 7G**). Among them, collagen family Col1a1, Col4a2 and Col5a1 are target genes of miR-29a-3p, and promote cancer cells invasion and migration (43–45). In addition, miR-29a can promote the neurite outgrowth by targeting extracellular matrix-related genes like Fibrillin 1 (Fbn1) and hyaluronan and proteoglycan link protein 1 (Hapln1) (46, 47), which dramatically increased in the pituitary of *miR-29a/b_1_* KO mice. Hdac4 (48), which is key epigenetic modified writer, may play important roles in the change of gene expression pattern in *miR-29a/b_1_* gene knockout mice, especially for down-regulated genes. For the 88 down-regulated proteins in the pituitary of *miR-29a/b_1_* KO mice, a considerable portion of them participate in vesicle-mediated transport and secretion (Ergic1, Fkbp2, Ssr3, Stat5a, Crhbp, **Figure 7F**). Notably, Ergic1, encodes a cycling membrane protein, and plays an important role in transport between endoplasmic reticulum and Golgi (49). Absence of trApγ (SSr3) impairs protein translocation into the endoplasmic reticulum and affects transport (50). Myosins were reported as core players in the final stages of regulated secretory pathways (51). Treatment of pituitary cells with the myosin light chain (Myl2/3) kinase inhibitor, wortmannin, attenuated GnRH-induced LH release (52). Further validated by quantitative PCR (qPCR) that the mRNA transcripts of these genes, which were consistent with LC-MS/MS (**Figure 7G**). These results indicated the deficiency of *miR-29a/b_1_* blocked proteins transportation, leading to impaired pituitary hormone secretion, especially LH released.

## Discussion

A lack of *miR-29a/b_1_* leads to female sterility in mice, which has been mentioned previously (1); however, the mechanisms underlying this result were not published or illustrated. In this work, we demonstrated that low serum LH level and ovulation disorder might be the direct cause of subfertility in female *miR-29a/b_1_* KO mice. This conclusion is further proved by the results that oocyte development is normal in the ovaries of mutant mice and normal eggs could be obtained through super-ovulated. Compared to wild-type mice, the pituitary gland in mutant mice stimulated with the same concentration of GnRH produced reduction LH secreted into the blood, indicating that *miR-29a/b_1_* KO females maintained normal pituitary responsiveness to GnRH, although expression of GnRHR was higher in *miR-29a/b_1_* KO females pituitaries which may represent compensation for plasma LH insufficiency (53). Meanwhile the expression of LH protein was higher in mutant pituitaries than that in wild types. This suggests that knockout of *miR-29a/b_1_* results in deficits in LH secretion from the pituitary but not in LH synthesis stimulated by GnRH (54, 55). Proteomic analysis of the pituitary showed that a large number of proteins related to cellular vesicle-mediated secretion and protein transport were significantly changed in *miR-29a/b_1_* KO mice. This effect seems to be omnidirectional, from the vesicle transport between endoplasmic reticulum and Golgi apparatus, as well as the process of docking and priming of secretory vesicle on the cell membrane. As a result, many kinds of secretory proteins, including LHβ, were accumulated in pituitary cells. These secreted proteins accounted for 44% of the upregulated proteins in the pituitary of mutant mice.

It is worth noting that FSH required for follicle growth and development and maturation of the ovum (56–58) was less affected by the knockout of *miR-29a/b_1_*. Different secretion modes between FSH and LH might be an important reason (59, 60). LH is secreted *via* a regulated pathway, while FSH release is primarily constitutive and controlled by synthesis. Increased FSH protein level in the mutant pituitary by 72% may compensate for the deficiency in the secretory mechanisms of the mutant mice (**Figure 7C**), which may also explain the fertility of male mutant mice. There is much agreement that FSH influences the mitotic activity of the spermatogonia and promote cellular differentiation during the pubertal phase (61). Testosterone regulated by LH also plays a role for spermatogenesis, however, completely T-independent spermatogenesis is possible if high-dose FSH treatment (62).

Of note, the use of intraventricular injection of *miR-29* inhibitor or overexpression of an antisense sequence targeting *miR-29* in the brain to knockdown expression of *miR-29* leads to earlier puberty onset or hyperfertility (63). These findings are not consistent with our results. It is possible that lack of *miR-29a/b_1_* function throughout development could result in compensatory effects which may lead to differences between our results and the results of the above literature. The underlying reasons for the different effects between knockout and knockdown need to be further studied. In addition, it should be noted that KO mice also showed growth retardation (64). We found that the weight of KO mice remained light, even though they had reached sexual maturity. So, the causal relation between two events cannot be confirmed now. We speculated that growth retardation and delayed maturity may come from the same reason, which happened in pituitary or upstream signal of KO mice.

In conclusion, LH secretion was impaired by *miR-29a/b_1_* knockout which caused ovulation deficiency in the mutant mice. Further studies revealed the effect of *miR-29a/b_1_* on hormone secretion function in the pituitary. Our work provides novel mechanistic insights into the relationship of *miR-29a/b_1_* and reproduction, opening the possibility of clinical approaches to reproductive studies based on the regulatory circuitry of *miR-29a/b_1_*.

## Data Availability Statement

The datasets presented in this study can be found in online repositories. The names of the repository/repositories and accession number(s) can be found in the article/**Supplementary Material**.

## Ethics Statement

The animal study was reviewed and approved by Institutional Animal Care and Use Committee of Shanghai Engineering Research Center for Model Organisms, SMOC. Written informed consent was obtained from the owners for the participation of their animals in this study.

## Author Contributions

YG, RSu, RSh and JF designed research. YG and JF analyzed data. YG, YW, HS, HZ, LC, QH, ZhiW, and YT performed research. YG, LX and JF wrote the paper. HY, MZ and ZhuW contributed to discussion and the proof reading of the paper. All authors contributed to the article and approved the submitted version.

## Funding

This work was supported by the grants from National Natural Science Foundation. of China (81261120568) and Science and Technology Commission of Shanghai Municipality (19DZ2280500, 18DZ2293500).

## Conflict of Interest

The authors declare that the research was conducted in the absence of any commercial or financial relationships that could be construed as a potential conflict of interest.

## Supplementary Material

Supplementary Table 1 List of oligonucleotides used.

Supplementary Figure 1 Genotyping of *miR-29a/b_1_* KO knockout mice. **(A)** The genotype of *miR-29a/b_1_* KO was identified by PCR amplification. There was 600bp deleted from the genomic DNA of *miR-29a/b_1_*. +/+: wild-type, +/-: heterozygous, -/-: homozygous. **(B)** Mature *miR-29a* RNA was detected in different tissues of wild-type mice but not in those of homozygous knockout mice (n=3). The precursor of *miR-29a* **(C)** and *miR-29b_1_* **(D)** RNA level was measured by quantitative RT-PCR in different tissues. *Pre-miR-29a* or *pre-miR-29b_1_* levels were decreased in *miR-29a/b_1_* ^+/-^ mice (n=10) and hardly detected in *miR-29a/b_1_^-/-^* mice (n=10) compared to wild-type littermates (n=8).

Supplementary Figure 2 Representative cell morphological changes in vaginal smears and estrous cycle pattern of four female mice in each wild-type and *miR-29a/b_1_* KO groups are shown.

Supplementary Figure 3
*miR-29a* expression patterns. **(A)** *miR-29a* expression patterns in wild-type mice during the estrous cycle in pituitary and ovary tissues (n=10). 1, proestrus; 2, estrus; 3, metestrus; 4, diestrus. **(B)** Relative expression levels of *miR-29a*, *miR-29b* and *miR-29c* in hypothalamus, pituitary and gonad (HPG axis) of wild-type and *miR-29a/b_1_* KO mice (♀miR-29c: hypothalamus: *p=0.6648*, pituitary: *p=0.4896*, ovary: *p=0.8028*, ♂miR-29c: hypothalamus: *p=0.0727*, pituitary: *p=0.1225*, testis: *p=0.5042*, n=5).

Supplementary Figure 4 Wet testis and seminal vesicle (SV) weight in males, normalized to body weight in the same animals (testis: wild-type: 0.7234 ± 0.02481, n=8, *miR-29a/b_1_*KO: 0.9563 ± 0.03834, n=9, *p=0.2107*; seminal: wild-type: 0.6085 ± 0.07193, n=8, *miR-29a/b_1_* KO: 0.5191 ± 0.03874, n=9, *p=0.2764*).

Supplementary Figure 5 Levels of selected hormones in females. **(A–C)** Serum FSH (wild-type: 12.23 ± 1.415, n=8, *miR-29a/b_1_* KO: 13.62 ± 1.255, n=13, *p=0.4850*) **(A)** and Testosterone (wild-type: 2.774 ± 0.1147, n=10, *miR-29a/b_1_* KO: 2.711 ± 0.2052, n=9, *p=0.7867*). **(B)** and Estradiol (wild-type: 207.9 ± 13.89, n=17, *miR-29a/b_1_* KO: 179.3 ± 15.54, n=19, *p=0.1834*). **(C)** of wild-type and *miR-29a/b_1_* KO mice were determined. **(D)** Expression of hormone synthesis-related gene in the ovaries in wild-type (n=4) and *miR-29a/b_1_* KO (n=10) females (*cyp11a*: *p=0.0159*, *cyp17a_1_*: *p=0.6094*, *cyp19a_1_*: *p=0.9604*).

## Abbreviations

miRNA, microRNA; iTRAQ, isobaric tags for relative and absolute quantification; PCR, polymerase chain reaction; LH, luteinizing hormone; FSH, follicle-stimulating hormone; KO, knockout.
