# Supplementary figures and images for: miR-29a/b1 Regulates the Luteinizing Hormone Secretion and Affects Mouse Ovulation
Source: Front Endocrinol (Lausanne). 2021 May 31;12:636220. doi: 10.3389/fendo.2021.636220 (PMC8202074; doi:10.3389/fendo.2021.636220)

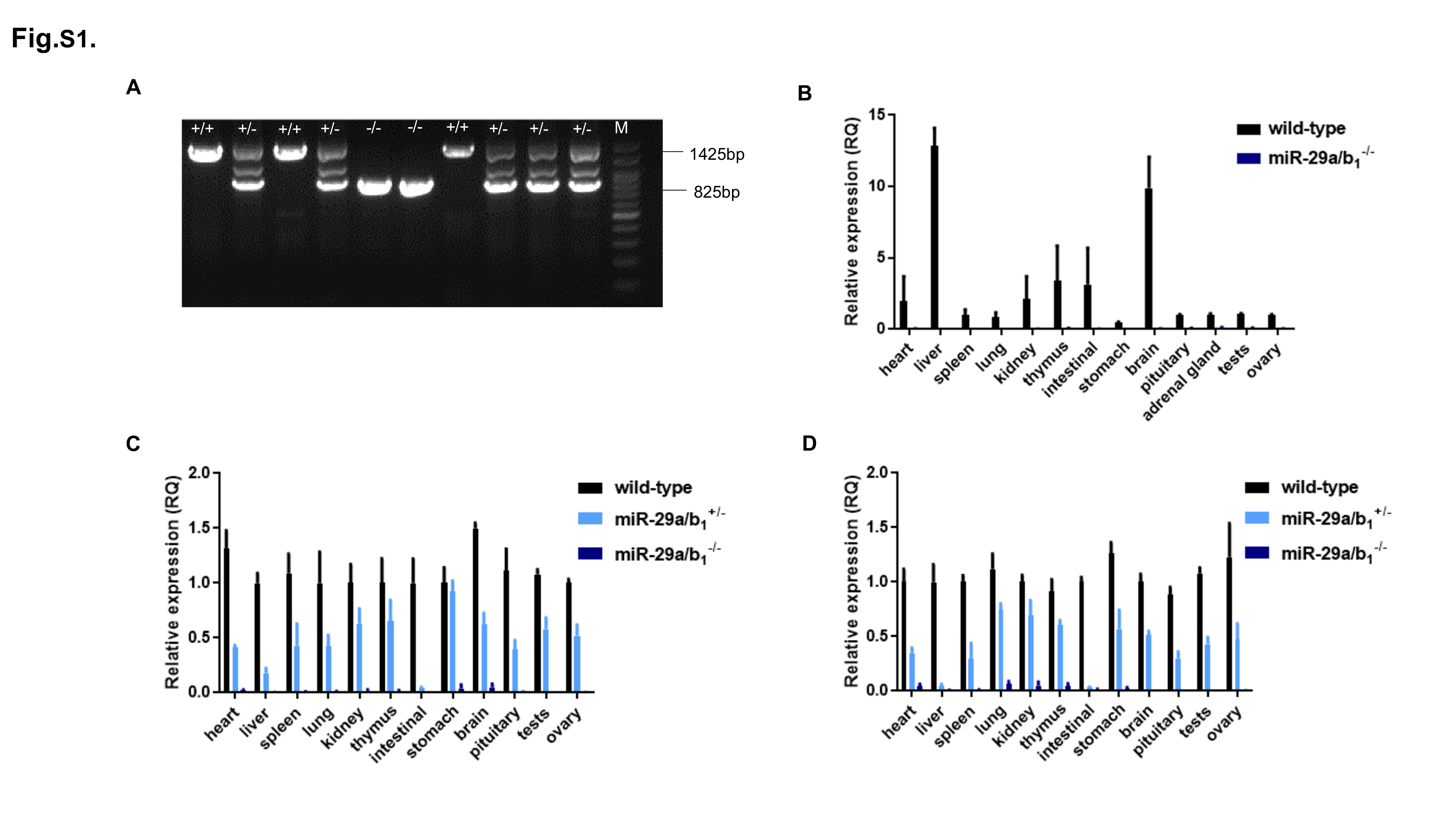

Supplement: Supplementary Figure 1 — Genotyping of miR-29a/b1 KO knockout mice. (A) The genotype of miR-29a/b1 KO was identified by PCR amplification. There was 600bp deleted from the genomic DNA of miR-29a/b1. +/+: wild-type, +/-: heterozygous, -/-: homozygous. (B) Mature miR-29a RNA was detected in different tissues of wild-type mice but not in those of homozygous knockout mice (n=3). The precursor of miR-29a (C) and miR-29b1 (D) RNA level was measured by quantitative RT-PCR in different tissues. Pre-miR-29a or pre-miR-29b1 levels were decreased in miR-29a/b1 +/- mice (n=10) and hardly detected in miR-29a/b1-/- mice (n=10) compared to wild-type littermates (n=8). [file DataSheet_1.zip › supplementary material/Fig S1.tif]

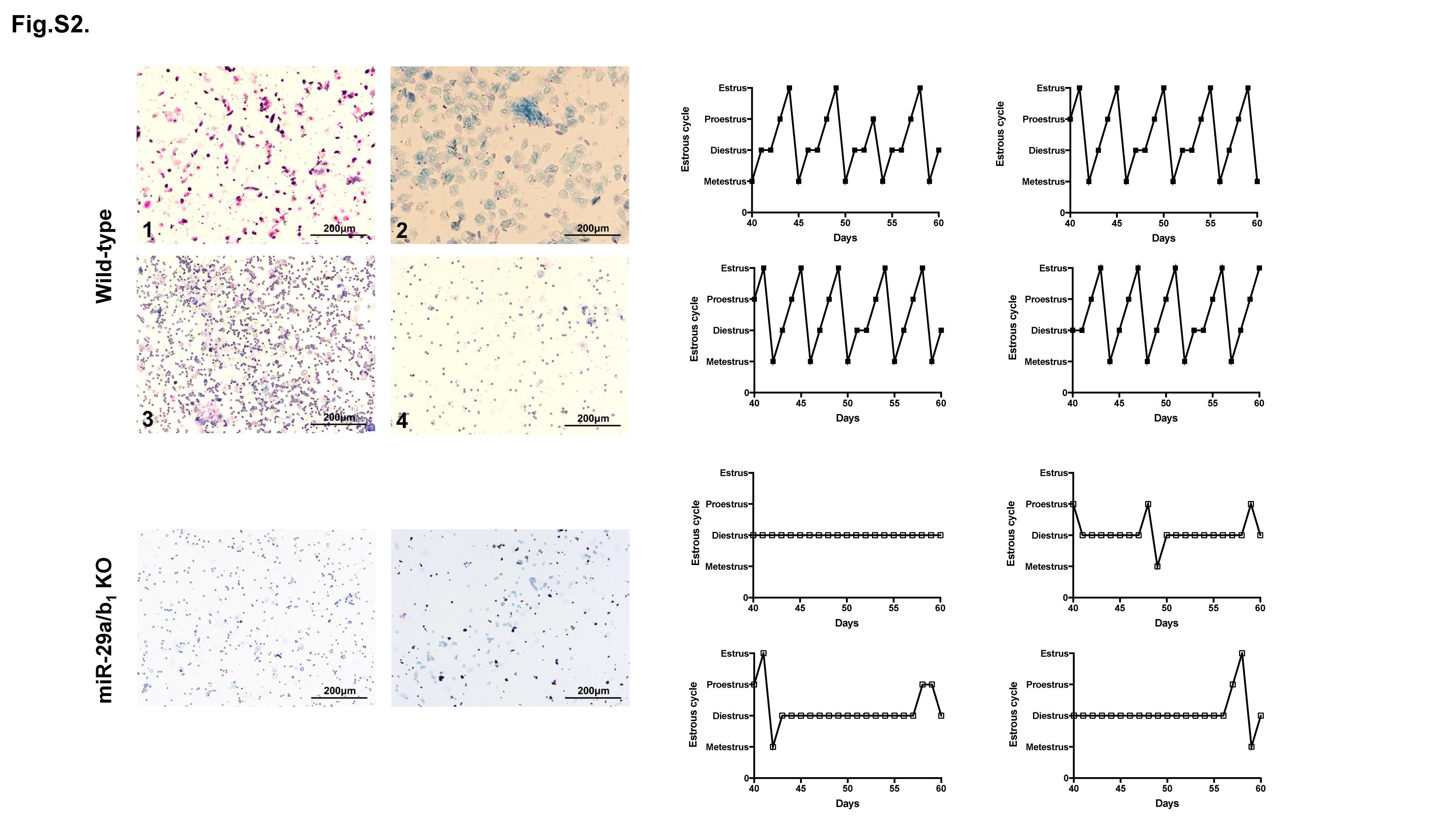

Supplement: Supplementary Figure 1 — Genotyping of miR-29a/b1 KO knockout mice. (A) The genotype of miR-29a/b1 KO was identified by PCR amplification. There was 600bp deleted from the genomic DNA of miR-29a/b1. +/+: wild-type, +/-: heterozygous, -/-: homozygous. (B) Mature miR-29a RNA was detected in different tissues of wild-type mice but not in those of homozygous knockout mice (n=3). The precursor of miR-29a (C) and miR-29b1 (D) RNA level was measured by quantitative RT-PCR in different tissues. Pre-miR-29a or pre-miR-29b1 levels were decreased in miR-29a/b1 +/- mice (n=10) and hardly detected in miR-29a/b1-/- mice (n=10) compared to wild-type littermates (n=8). [file DataSheet_1.zip › supplementary material/Fig. S2.tif]

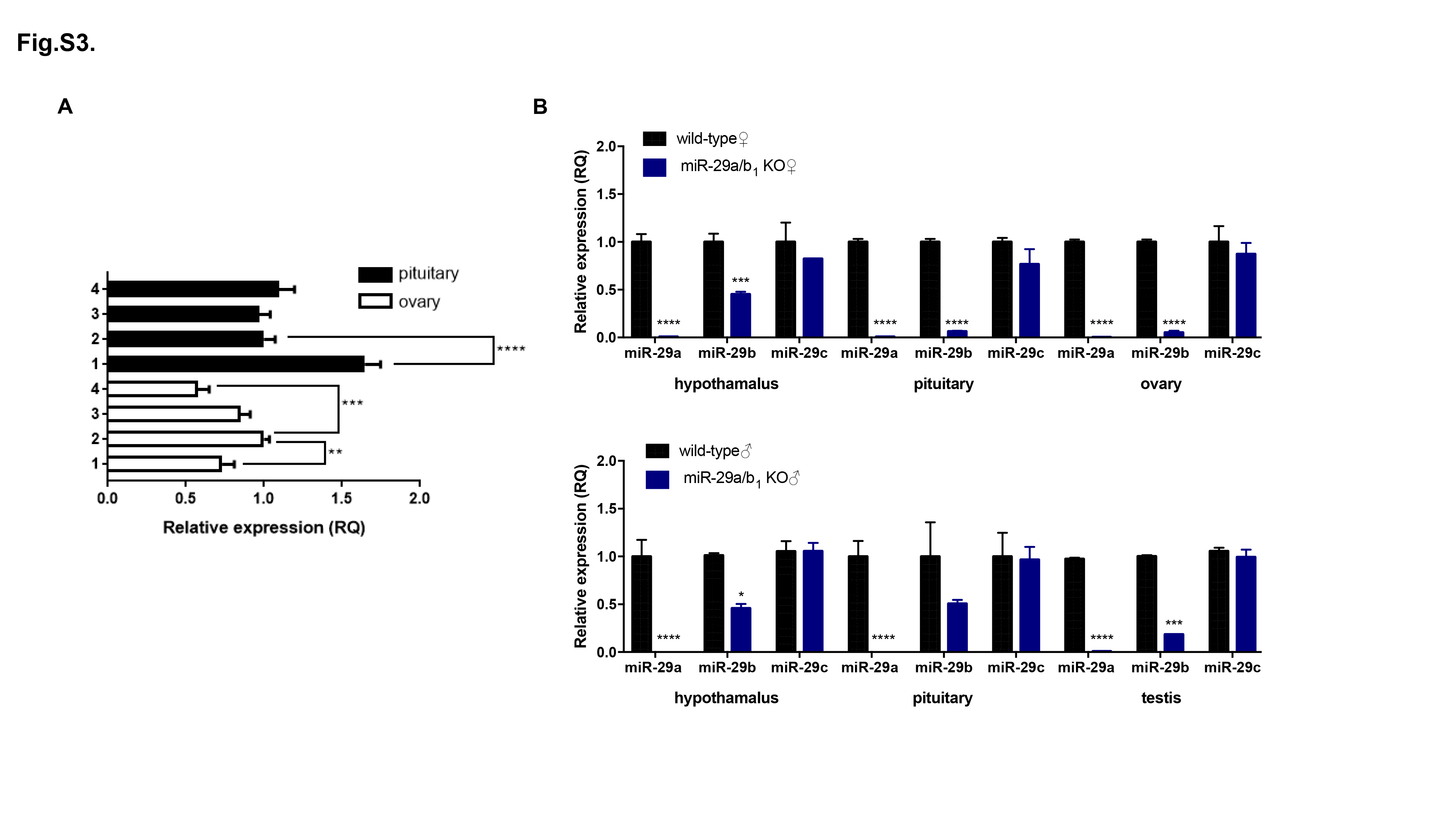

Supplement: Supplementary Figure 1 — Genotyping of miR-29a/b1 KO knockout mice. (A) The genotype of miR-29a/b1 KO was identified by PCR amplification. There was 600bp deleted from the genomic DNA of miR-29a/b1. +/+: wild-type, +/-: heterozygous, -/-: homozygous. (B) Mature miR-29a RNA was detected in different tissues of wild-type mice but not in those of homozygous knockout mice (n=3). The precursor of miR-29a (C) and miR-29b1 (D) RNA level was measured by quantitative RT-PCR in different tissues. Pre-miR-29a or pre-miR-29b1 levels were decreased in miR-29a/b1 +/- mice (n=10) and hardly detected in miR-29a/b1-/- mice (n=10) compared to wild-type littermates (n=8). [file DataSheet_1.zip › supplementary material/Fig.S3.tif]

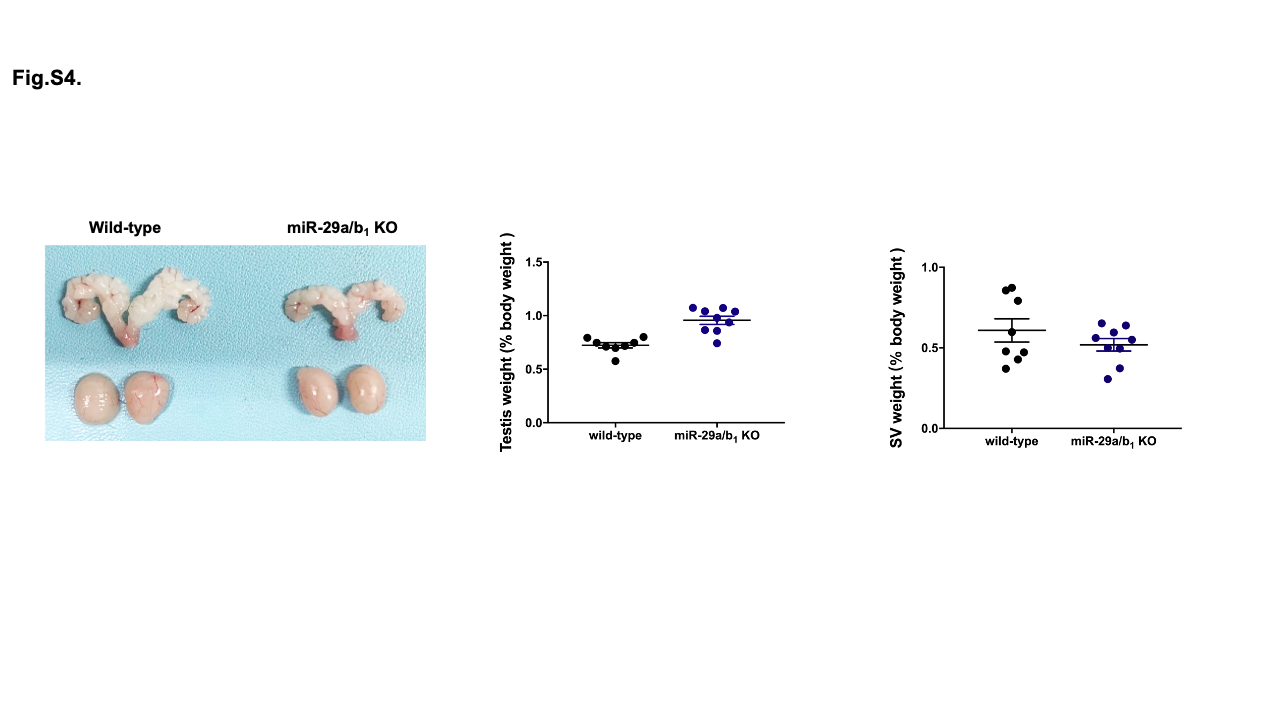

Supplement: Supplementary Figure 1 — Genotyping of miR-29a/b1 KO knockout mice. (A) The genotype of miR-29a/b1 KO was identified by PCR amplification. There was 600bp deleted from the genomic DNA of miR-29a/b1. +/+: wild-type, +/-: heterozygous, -/-: homozygous. (B) Mature miR-29a RNA was detected in different tissues of wild-type mice but not in those of homozygous knockout mice (n=3). The precursor of miR-29a (C) and miR-29b1 (D) RNA level was measured by quantitative RT-PCR in different tissues. Pre-miR-29a or pre-miR-29b1 levels were decreased in miR-29a/b1 +/- mice (n=10) and hardly detected in miR-29a/b1-/- mice (n=10) compared to wild-type littermates (n=8). [file DataSheet_1.zip › supplementary material/Fig.S4.tiff]

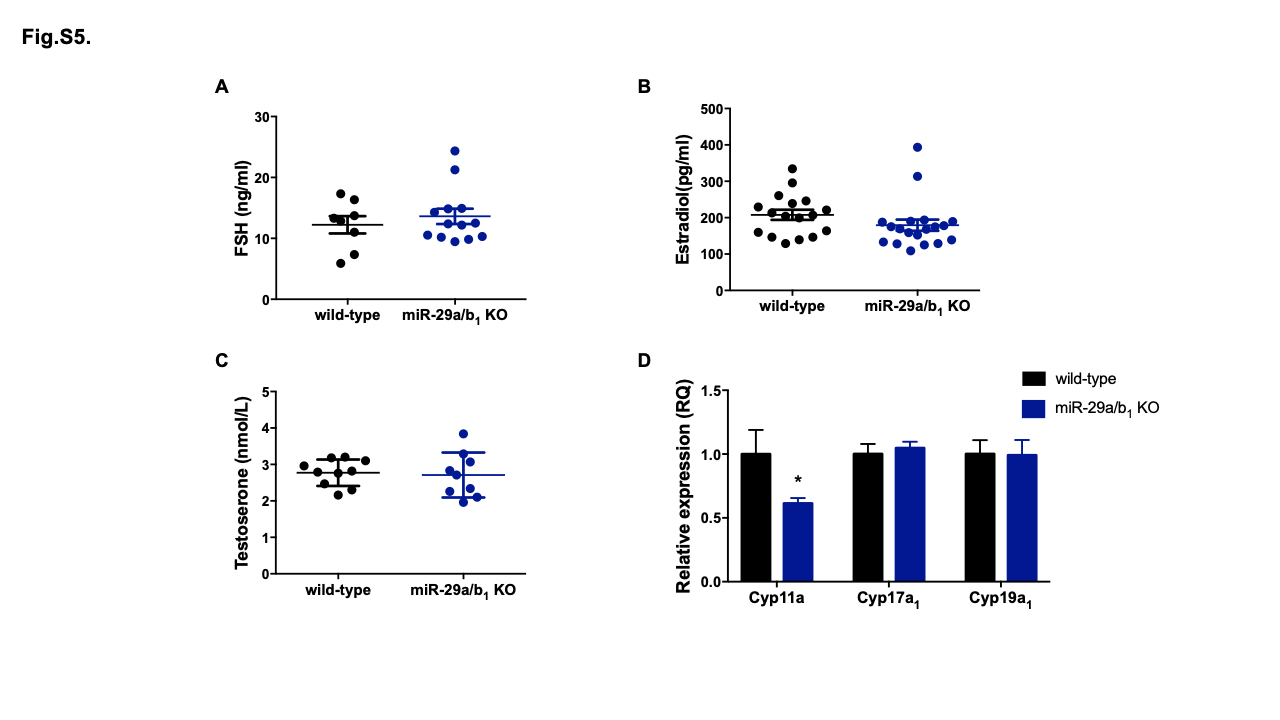

Supplement: Supplementary Figure 1 — Genotyping of miR-29a/b1 KO knockout mice. (A) The genotype of miR-29a/b1 KO was identified by PCR amplification. There was 600bp deleted from the genomic DNA of miR-29a/b1. +/+: wild-type, +/-: heterozygous, -/-: homozygous. (B) Mature miR-29a RNA was detected in different tissues of wild-type mice but not in those of homozygous knockout mice (n=3). The precursor of miR-29a (C) and miR-29b1 (D) RNA level was measured by quantitative RT-PCR in different tissues. Pre-miR-29a or pre-miR-29b1 levels were decreased in miR-29a/b1 +/- mice (n=10) and hardly detected in miR-29a/b1-/- mice (n=10) compared to wild-type littermates (n=8). [file DataSheet_1.zip › supplementary material/Fig S5.tiff]
